# Division site positioning in bacteria: one size does not fit all

**DOI:** 10.3389/fmicb.2014.00019

**Published:** 2014-02-03

**Authors:** Leigh G. Monahan, Andrew T. F. Liew, Amy L. Bottomley, Elizabeth J. Harry

**Affiliations:** The ithree Institute, University of TechnologySydney, NSW, Australia

**Keywords:** cell division, ftsZ, min system, nucleoid occlusion, Z ring, division regulation, bacterial cell division

## Abstract

Spatial regulation of cell division in bacteria has been a focus of research for decades. It has been well studied in two model rod-shaped organisms, *Escherichia coli* and *Bacillus subtilis*, with the general belief that division site positioning occurs as a result of the combination of two negative regulatory systems, Min and nucleoid occlusion. These systems influence division by preventing the cytokinetic Z ring from forming anywhere other than midcell. However, evidence is accumulating for the existence of additional mechanisms that are involved in controlling Z ring positioning both in these organisms and in several other bacteria. In some cases the decision of where to divide is solved by variations on a common evolutionary theme, and in others completely different proteins and mechanisms are involved. Here we review the different ways bacteria solve the problem of finding the right place to divide. It appears that a one-size-fits-all model does not apply, and that individual species have adapted a division-site positioning mechanism that best suits their lifestyle, environmental niche and mode of growth to ensure equal partitioning of DNA for survival of the next generation.

## INTRODUCTION

The location of proteins inside bacterial cells is tightly regulated, with different proteins having specific cellular addresses that can change dynamically with time (Shapiro et al., 2009; Rudner and Losick, 2010; Lenz and Sogaard-Andersen, 2011; Nevo-Dinur et al., 2012). This exquisite spatial organization has provided unique mechanistic insights into fundamental biological processes. Cell division, or cytokinesis, is one such process that is under strict spatial control and this ensures equal partitioning of DNA into newborn cells (Thanbichler, 2009; Lutkenhaus et al., 2012; Monahan and Harry, 2013). Just how bacterial cells identify the site of division is an important question that has not been fully resolved.

The first protein to localize to the division site in bacteria is the highly conserved tubulin-like protein, FtsZ. FtsZ polymerizes at this site to form a ring, called the Z ring, which then recruits all other known division proteins, about 20 in all, to form a protein complex called the divisome (Adams and Errington, 2009; De Boer, 2010). The divisome subsequently contracts, pulling in the cell envelope to facilitate cytokinesis. Importantly, the Z ring marks the site of cell division. Not surprisingly therefore, proteins that are known to influence division site placement within bacterial cells, actually do so via their influence on the position of the Z ring.

## NUCLEOID OCCLUSION AND THE MIN SYSTEM

In the model rod-shaped bacteria, *Escherichia coli* and *Bacillus subtilis*, Z ring formation occurs precisely at the cell midpoint (standard deviation of 2.6 and 2.2% off center, respectively; Yu and Margolin, 1999; Migocki et al., 2002), generating daughter cells of equal size. Research into the control of Z ring positioning in these organisms has centered mainly on two regulatory systems, nucleoid occlusion (NO) and the Min system, which inhibit Z rings forming anywhere in the cell other than midcell.

**FIGURE 1:**
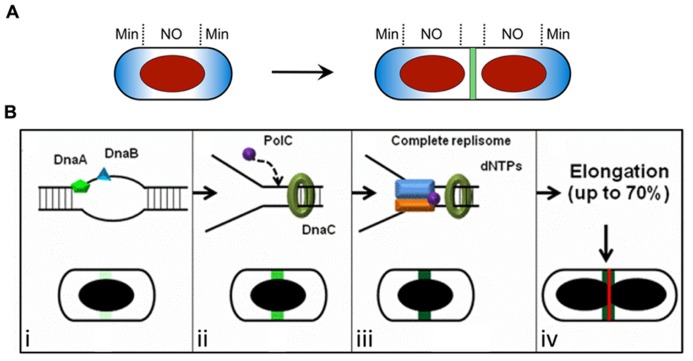
**Spatial regulation of Z ring assembly in *E. coli* and *B. subtilis*.** **(A)** The Min system and nucleoid occlusion (NO) inhibit Z ring formation at inappropriate sites. The Min proteins (blue) are concentrated to the cell poles, while the nucleoid (red) occupies the central region of the cell. The later stages of chromosome segregation result in a relief of nucleoid occlusion due to the removal of Noc/SlmA away from midcell, allowing Z ring formation at this site. **(B)** The “Ready-Set-Go” model proposes that identification of the division site is linked to the progress of DNA replication in *B. subtilis*, independently of both the Min system and nucleoid occlusion (Moriya et al., 2010). (i–iv) Progression of the initiation phase of DNA replication in *B. subtilis*, resulting in replisome assembly at *oriC*, promotes the maturation of a midcell site. This may occur due to the accumulation of a factor at midcell that activates FtsZ polymerization into a ring at this site (increasing darkness of green shading). (i) The binding of the early initiation protein DnaA to unwind the DNA at *oriC* starts midcell “potentiation.” Next, other early DNA replication initiation proteins, such as DnaB, bind this chromosomal region and increase midcell potentiation (light green area at midcell). (ii) DnaC helicase is then loaded, followed by PolC (the α-subunit of DNA polymerase III) and other replisome components, creating the replication fork at *oriC*. This further potentiates midcell (green area at midcell). (iii) Assembly of the remaining replisome components to complete initiation, ready for DNA synthesis, allows 100% potentiation of midcell (dark green area at midcell) for Z ring formation (red line). (iv) Midcell Z ring formation does not occur straight away since this requires ~70% of the chromosome to be replicated (Wu et al., 1995) to clear the bulk of the replicating DNA from midcell. **(A)** is reproduced from Monahan and Harry (2013) and **(B)** is adapted from Moriya et al. (2010), both with permission from Wiley Interscience.

The Min system blocks Z ring assembly at the cell poles by inhibiting the polymerization of FtsZ (Barak and Wilkinson, 2007; Lutkenhaus, 2007; Bramkamp and van Baarle, 2009). In both *E. coli* and *B. subtilis*, the MinC protein serves as the primary inhibitor of Z ring formation by direct interaction and destablization of FtsZ polymers (Hu et al., 1999; Dajkovic et al., 2008). MinC is localized to the cytoplasmic membrane via association with the membrane-bound ATPase MinD, and is directed to the poles by fundamentally different mechanisms in *E. coli* and *B. subtilis*. In *B. subtilis* DivIVA pilots MinCD to the poles via the bridging protein MinJ (Bramkamp et al., 2008; Patrick and Kearns, 2008), while in *E. coli* the topological determinant MinE undergoes a dynamic pole-to-pole oscillation that actively displaces the MinCD complex from the membrane. The net result in both organisms is that the MinCD concentration is highest in the polar regions of the cell, blocking polar Z ring formation (**Figure 1A**).

Nucleoid occlusion prevents Z ring assembly over the nucleoid or chromosome (Wu and Errington, 2012). This effect is mediated at least in part by the non-homologous proteins Noc in *B. subtilis* (Wu and Errington, 2004; Wu et al., 2009) and SlmA in *E. coli* (Bernhardt and de Boer, 2005; Cho et al., 2011; Tonthat et al., 2011; Tonthat et al., 2013), which localize over nucleoids by binding to specific DNA sequences. SlmA affects FtsZ polymerization directly (Cho et al., 2011; Tonthat et al., 2011), while it is unclear how Noc influences Z ring formation (Wu and Errington, 2012). Interestingly, binding sites for both Noc and SlmA are sparse or absent near the terminus region of the chromosome (Wu et al., 2009; Cho et al., 2011; Tonthat et al., 2011), which occupies a midcell location during the late stages of chromosome replication and segregation. Thus chromosome segregation generates a relief of NO at midcell, allowing the Z ring to form there (**Figure 1A**).

## A LINK BETWEEN DNA REPLICATION AND Z RING POSITIONING

There is no doubt that in *E. coli* and *B. subtilis* Min and NO play an important role in influencing Z ring placement (**Figure 1A**; Rothfield et al., 2005; Harry et al., 2006; Barak and Wilkinson, 2007; Wu and Errington, 2012). However, there are now several lines of evidence that strongly implicate additional Z ring positioning mechanisms in these rod-shaped bacteria. First, cells are still viable in the absence of both the Min system and NO proteins, Noc/SlmA, although viability is conditional and division is much less efficient (Wu and Errington, 2004; Bernhardt and de Boer, 2005; Rodrigues and Harry, 2012). Second, overproduction of FtsZ in *min* *noc* *B. subtilis* and *min* *slmA* *E. coli* double mutants results in partial restoration of division, with Z rings assembling at the correct site between segregated chromosomes (Wu and Errington, 2004; Bernhardt and de Boer, 2005).

More recently, it was shown using outgrown spores of *B. subtilis* that Z rings can form precisely at midcell in this organism in the complete absence of both the Min system and Noc (Rodrigues and Harry, 2012). In these *min* *noc* outgrown cells, Z rings were positioned at midcell with wild-type precision, although their assembly was delayed and less efficient, with Z rings forming also at future division sites (1/4 and 3/4 positions) and the cell poles (Rodrigues and Harry, 2012). Overproduction of FtsZ in these cells significantly reduced the delay, and increased the proportion of midcell Z rings (Rodrigues and Harry, 2012). To test whether any NO is required for precise positioning of the Z ring at midcell, chromosomes in *min noc* outgrown cells were allowed to replicate and separate to the extent that a significant gap of DNA (no NO) occurred in the central region of the cell. FtsZ production was then switched on (Rodrigues and Harry, 2012). Remarkably, Z rings formed precisely at midcell under these conditions, and there was a high preference (87%) for Z ring assembly at midcell as opposed to any other DNA-free regions, including the cell poles (Rodrigues and Harry, 2012).

The above data argue for the identification of a specific site at midcell for Z ring assembly in *B. subtilis* that does not require Min or any NO, and have led to a model in which NO and Min do not identify the correct division site at midcell but rather ensure that the Z ring forms there and only there, at the right time in the cell cycle (Rodrigues and Harry, 2012). In other words, in *B. subtilis* at least, Min and NO do not appear to be the division “signpost,” but enable this signpost at midcell to be efficiently utilized.

So what does identify the division site? In *B. subtilis* it has been proposed that a positive signal links progress of the initiation phase of DNA replication with identification of the division site at midcell (**Figures 1A,B**; Moriya et al., 2010; Rodrigues and Harry, 2012). It has been known for some time that the early stages of DNA replication influence Z ring positioning in this organism (Harry et al., 1999; Regamey et al., 2000). More recently it has been shown that even in the absence of Noc the frequency of midcell Z rings increases with progression of the initiation phase of replication (Moriya et al., 2010). This has led to a model for Z ring positioning in *B. subtilis*, called the “Ready-Set-Go” model, in which midcell becomes increasingly “potentiated” for Z ring formation as initiation of DNA replication is progressively completed, much like a runner in a race changing position in readiness to race from the start line when the gun fires (**Figure 1B**; Moriya et al., 2010). The ordered, stable association of replication initiation proteins with *oriC* in *B. subtilis* (as opposed to all initiation proteins associating with *oriC* simultaneously) is consistent with a progressive step-wise potentiation of midcell (Smits et al., 2010). Importantly the observation that Z rings can form precisely at midcell even when there is no DNA synthesis (elongation) establishes that the midcell site is determined in *B. subtilis* very early in the cell cycle, much earlier than when Z ring formation actually occurs. However, its utilization is blocked [either by a NO protein other than Noc (Bernard et al., 2010) or some other mechanism] until the chromosome has been replicated beyond 70% completion (**Figure 1B**; Wu et al., 1995).

In *E. coli*, SlmA-, MinC-, and SOS-independent inhibition of midcell Z rings can occur over a partially replicated, unsegregated nucleoid indicating that additional Z-ring positioning mechanisms also exist in this organism (Bernard et al., 2010; Cambridge et al., 2013). Interestingly, the localization of the replisome protein, DnaX, to midcell prior to *oriC* and the Z ring in *E. coli* raises the possibility that DNA replication has a positive role in division site positioning in organisms other than *B. subtilis* (Bates and Kleckner, 2005).

## CONTROL OF Z RING PLACEMENT IN OTHER ORGANISMS: A DIVERSITY OF MECHANISMS

The importance of Z ring positioning mechanisms other than Min and NO is further exemplified by the fact that many bacteria lack Noc/SlmA and/or Min protein homologues (Margolin, 2005; Harry et al., 2006). Recent studies on division site selection in several such bacteria have uncovered a number of novel Z-ring positioning mechanisms. These include both negative regulators (FtsZ inhibitors) as well as positive signals that actively promote Z ring formation at the correct location. Interestingly, many of the proteins involved in these systems are not highly conserved, suggesting that Z ring placement is controlled differently between different bacterial species. Cell shape is also emerging as an important factor in division site selection, particularly in spherical cells that must select the correct division plane from a theoretically infinite number of possible midcell planes (Margolin, 2000). Different coccal species select different midcell planes for division (*Staphylococci* divide in three alternating planes and *Neisseria* in two for example), further highlighting the species-specific nature of bacterial division site placement. Below we describe the different positioning mechanisms at play in a range of recently studied organisms.

### *Caulobacter crescentus*: MipZ

**FIGURE 2:**
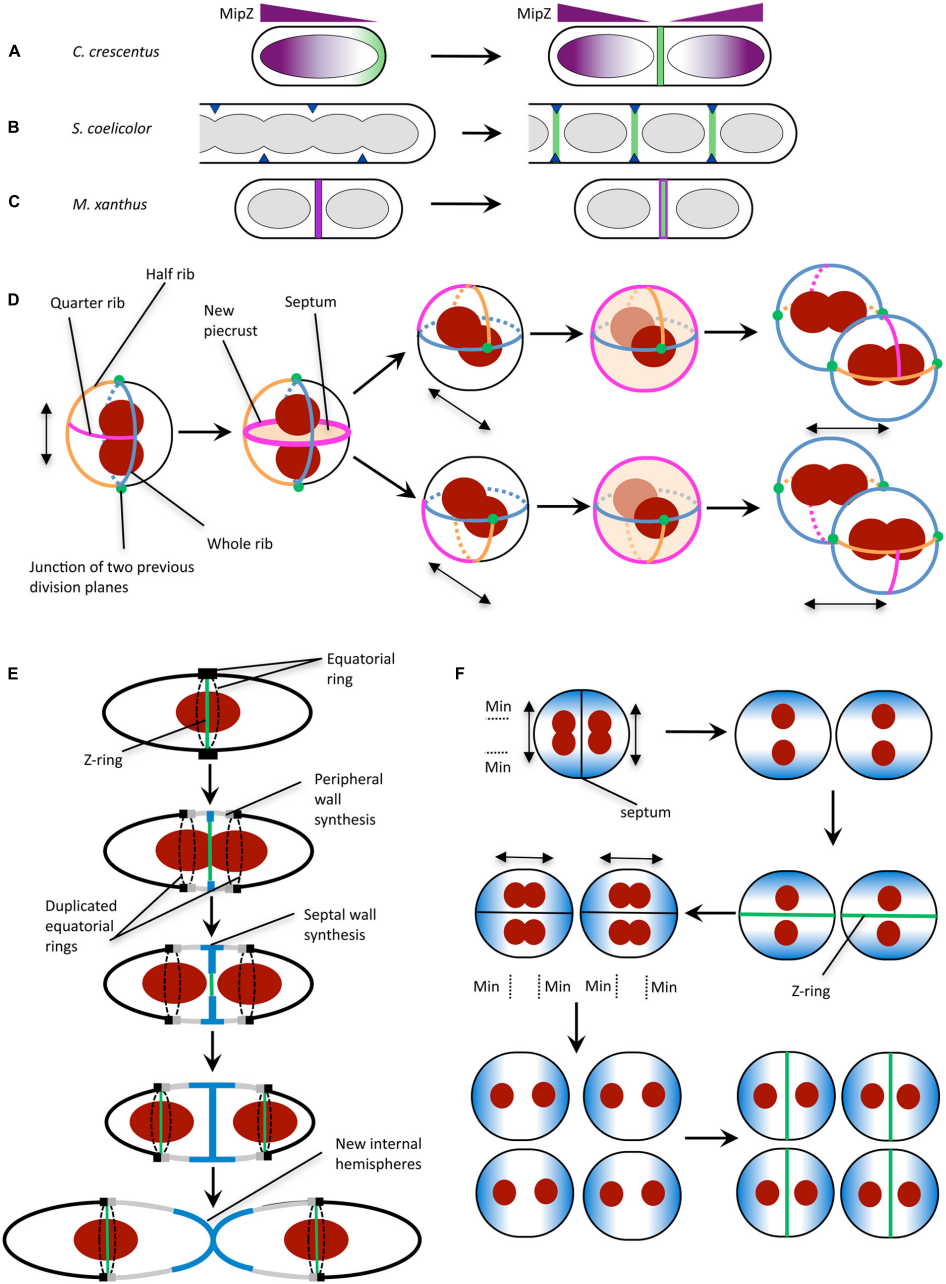
**Mechanisms for division site selection in different bacterial species.** **(A)** In *C. crescentus,* Z ring positioning is controlled by the FtsZ inhibitor MipZ (purple). MipZ associates with the chromosome, and forms a gradient of decreasing concentration with distance from the cell pole (see text). In newborn cells with one chromosome, the MipZ gradient is established from a single pole, confining FtsZ (green) to the opposite pole. Chromosome replication and segregation establishes a bipolar MipZ gradient that dislodges FtsZ from the pole and restricts Z ring assembly to midcell. **(B)** In sporulating hyphae of *S. coelicolor,* Z ring assembly is positively regulated by the SsgB protein (dark blue), which localizes to division sites (via SsgA) then recruits FtsZ (green). SsgB remains associated with the Z ring. **(C)** In *M. xanthus,* PomZ (magenta) localizes to the cell center following chromosome segregation, then recruits FtsZ to form the Z ring (green). **(D)** *S. aureus* has been proposed to utilize specific peptidoglycan features in conjunction with nucleoid occlusion for division in three perpendicular planes. Double-headed arrow indicates the axis of chromosome segregation. The plane for septum formation is marked by the presence of the quarter rib. The axis of chromosome segregation is determined by the movement of the nucleoids to junctions of two previous division planes. To determine the next division plane, Staphylococcal cells may recognize the quarter rib feature via a direct receptor-ligand type interaction (Turner et al., 2010). The plane containing the quarter rib also has the longest circumference which could be used for recognition by the cell (Turner et al., 2010). Division plane selection might also be aided by establishing the axis of chromosome segregation toward the junctions between division planes (green circles). **(E)** In *S. pneumoniae,* division plane selection has been suggested to rely on the presence of equatorial rings for cell division in consecutive parallel planes (see text). Equatorial rings are present at the cell equator and mark the site for septal cell wall synthesis through recruitment of divisome components such as FtsZ. The equatorial rings are then duplicated and move apart, via the synthesis of new peripheral peptidoglycan, until both rings are located at the equators of the new daughter cells. New peripheral and septal peptidoglycan synthesis are highlighted in gray and blue, respectively. **(F)** *N. gonorrhoeae* cell division in alternating perpendicular planes. At the onset of cell division, two temporarily asymmetric daughter cells are generated which have a short and long axis (Pinho et al., 2013). This leads to the oscillation of the Min protein complex as well as chromosome segregation along the long axis, which is parallel to the septal plane (Pinho et al., 2013). Note that *N. gonorrhoeae* does not contain a Noc/SlmA homolog, but the presence of the replisome machinery around the DNA may negatively regulate divisome assembly around the DNA, analogous to the action of Noc (Ramirez-Arcos et al., 2002). Double-headed arrow indicates axis of chromosome segregation. **(A)** is reproduced from Monahan and Harry (2013) and **(B,C)** are adapted from Monahan and Harry (2013) with permission from Wiley Interscience.

In *Caulobacter crescentus*, which lacks both Min and NO proteins, Z ring positioning is governed by a bipolar gradient of the FtsZ inhibitor MipZ (**Figure 2A**; Thanbichler and Shapiro, 2006). Interestingly, MipZ belongs to the same family of ATPases as MinD, but unlike MinD it acts on FtsZ directly to block Z ring assembly (Thanbichler and Shapiro, 2006). MipZ is conserved across all α-proteobacteria that lack MinCD orthologs.

The bipolar MipZ gradient relies on interaction with the chromosome partitioning protein ParB, which itself localizes to cell poles via association with the chromosomal origin region. Interaction with ParB triggers the formation of MipZ dimers, which diffuse away and bind non-specifically to the chromosome (Kiekebusch et al., 2012). This establishes a gradient of decreasing MipZ concentration with increasing distance from the cell pole (the location of ParB). The intrinsic ATPase activity of MipZ releases MipZ monomers from the chromosome, which are then re-captured by ParB to continue the cycle (Kiekebusch et al., 2012).

Importantly, MipZ provides both spatial and temporal cues for Z ring formation. In newborn *C. crescentus* cells, which contain only one chromosomal origin, the MipZ gradient is established from a single pole, restricting FtsZ to the opposite pole (**Figure 2A**). Replication and segregation of the chromosomal origins then sets up a bipolar gradient in pre-divisional cells, which dislodges FtsZ from the pole and restricts Z ring formation to midcell (**Figure 2A**).

### *Streptomyces coelicolor*: SsgAB

In sporulating cells of *Streptomyces coelicolor*, which also lacks the Min and NO systems, FtsZ is recruited and tethered to the division site directly via interaction with the membrane-associated protein SsgB (**Figure 2B**; Willemse et al., 2011). SsgB promotes FtsZ polymerization *in vitro*, and presumably stimulates Z ring assembly in *S. coelicolor* cells. The localization of SsgB is mediated by the orthologous protein SsgA (Traag and van Wezel, 2008; Willemse et al., 2011). Significantly, the SsgAB system was the first positive control mechanism to be reported for Z ring positioning (Willemse et al., 2011). However, both SsgA and SsgB are only present in *Actinomycetes*.

### *Myxococcus xanthus*: PomZ

Another positive regulatory mechanism for Z ring placement has recently been reported in *Myxococcus xanthus*, a rod-shaped δ-proteobacterium that lacks all of the known FtsZ positioning proteins. In this organism, a novel protein called PomZ was shown to be required for efficient Z ring formation and for midcell Z ring placement (Treuner-Lange et al., 2013). Importantly, PomZ localizes to the division site, and does so both prior to and independently of FtsZ (Treuner-Lange et al., 2013). On this basis, it has been suggested that PomZ may not only identify the division site, but recruit FtsZ to this site and stabilize the Z ring (**Figure 2C**; Monahan and Harry, 2013; Treuner-Lange et al., 2013). Indeed, PomZ was shown to pull down FtsZ from whole-cell *M. xanthus* extracts (Treuner-Lange et al., 2013). However, it is not yet clear whether the two proteins bind directly or via an interaction partner.

### Staphylococcus aureus

*Staphylococcus aureus* divides sequentially in orthogonal planes in three dimensional space (Tzagoloff and Novick, 1977), raising the question of how staphylococcal cells can re-orientate the division machinery and how these daughter cells then “remember” previous division events for subsequent divisions. Interestingly, *S. aureus* lacks a visible Min homolog but does contain a homolog of the nucleiod occlusion protein, Noc (Veiga et al., 2011). Noc co-localizes with the nucleoid and reduces the frequency of Z-ring formation over DNA (Veiga et al., 2011), consistent with its role in *B. subtilis* (Wu and Errington, 2004). The nucleoid in *S. aureus* fills a large region of the cytoplasmic space (Yu et al., 2010), which suggests that nucleiod occlusion plays a critical role in division plane selection (Pinho et al., 2013).

In order to divide in more than one plane with fidelity however, *S. aureus* needs to carry information from previous planes of division. This data is spatially affected by each round of division and so is unlikely to be encoded by DNA. It is possible that non-DNA cell components, such as the cell wall (Turner et al., 2010) spatially regulate division site selection by acting as a marker for previous and potential division planes. Early studies of *S. aureus* cell wall architecture revealed a heterogeneity in cell wall thickness (Gilbo et al., 1967; Touhami et al., 2004) which have been more recently defined as equatorial rings of thicker bands of peptidoglycan (“piecrusts”), as well as orthogonal bands of peptidoglycan which presumably represent remnants of previous piecrust features (**Figure 2D**; Turner et al., 2010). These differences in cell wall architecture may encode epigenetic information in *S. aureus* (that could be recognized by currently unidentified protein components; Veiga et al., 2011) to maintain planar division site selection with fidelity over many generations due to the presentation of specific cell wall architectural features denoting the division plane from two previous rounds of division (Turner et al., 2010).

Negative regulation of midcell selection by cell wall components may also play a role; a cryo-electron microscopy study revealed that wall teichoic acids (WTAs) may not be uniformly localized throughout the cell wall, at least during septum formation (Matias and Beveridge, 2007). WTA localization has been shown previously to affect the midcell localization of Atl and PBP4 in *S. aureus* (Atilano et al., 2010; Schlag et al., 2010) indicating that WTA distribution could regulate midcell localization. However, the role of cell wall architecture and composition in division site selection, and the mechanisms by which bacteria translate this architectural information to ensure correct division site selection still remains elusive.

### Streptococcus pneumoniae

Ovococci, similar to rod-shaped organisms, divide in a single plane along the short axis of the cells (Margolin, 2000). However, the genomes of Streptococci and Enterococci show an absence of Noc and Min homologs (Pinho et al., 2013). The lack of NO is further illustrated by the observation that Z-rings in *S. pneumoniae* frequently form over nucleoids and that cell constriction occurs concurrently with the separation of the DNA (Land et al., 2013). Cell division in Streptococci begins with divisome assembly and initial in-growth of the septum at the cell equator which is marked by the equatorial ring (Higgins and Shockman, 1970; Wheeler et al., 2011). Soon after, this equatorial ring is duplicated and moves apart due to nascent peptidoglycan insertion between the rings. This “peripheral” elongation continues until new internal hemispheres are formed (Massidda et al., 2013). Presumably, the presence of the equatorial rings (analogous to the “piecrust” features seen in *S. aureus*; Wheeler et al., 2011) in the daughter cells alone (**Figure 2E**) can serve as a marker for the cell equator and hence the site for future cell division (Zapun et al., 2008) through an as yet-unidentified mechanism.

### Neisseria gonorrhoeae

*Neisseria gonorrhoeae* (Ng) is a Gram-negative coccus that divides in two alternating perpendicular planes (Westling-Häggström et al., 1977) resulting in a tetrad of daughter cells. *N. gonorrhoeae* lacks a Noc or SlmA homolog but encodes MinC, MinD, and MinE. In contrast to *E. coli*, deletion of *minC*_Ng_ (Ramirez-Arcos et al., 2001) as well as *minD*_Ng_ (Szeto et al., 2001) led to abnormal cell division, lysis and reduced viability of gonococcal cells highlighting their importance for cell division in this organism. Furthermore, heterologous production of a GFP-tagged MinD_Ng_ in round *E. coli rodA* mutants showed GFP-MinD_Ng_ oscillating along the axis parallel to the septa (Ramirez-Arcos et al., 2002). This oscillation pattern along the long axis of the cell could generate a MinCD inhibitory concentration gradient which is lowest in the plane that is perpendicular to a previous division event thereby allowing septum formation within the next plane (**Figure 2F**; Ramirez-Arcos et al., 2002; Pinho et al., 2013).

## CONCLUSION AND PERSPECTIVES

While there are some recurring themes for the spatial regulation of cell division in bacteria, such as the use of the ParA/MinD family of ATPases in several organisms (Lutkenhaus, 2012; Treuner-Lange et al., 2013), many bacteria do it differently, with the proteins and mechanisms being less conserved than was once thought. The ultimate purpose of division site regulation is to ensure that cytokinesis produces viable daughter cells. The diversity of mechanisms by which bacteria can achieve this is further exemplified by a recent study of *Mycobacterium* spp., which showed that Z ring positioning is essentially random in these organisms and that cell division can occur over nucleoids. This appears to be compensated by post-septal DNA transport rather than strict control of division site placement (Singh et al., 2013).

There are several factors that might have enabled bacteria to evolve different positioning mechanisms. These include cell shape (e.g., rod versus coccus), mode of peptidoglycan synthesis (elongation/division modes), lifestyle (division occurs asymmetrically in *C. crescentus* for example, and is only essential during sporulation in *Streptomyces*), as well as the repertoire of FtsZ-binding proteins present in the organism. A striking recent example of niche-specific division site selection comes from the study of a rod-shaped γ-proteobacterium that adheres via one of its poles to the nematode *Laxus oneistus*. To ensure that both daughter cells remain attached to the host, this bacterium grows by increasing in width and forms its septum parallel rather than perpendicular to the length axis (Leisch et al., 2012). We are likely to find more variations on the division-positioning mechanism as more bacterial species are examined. Establishing the mechanistic details of these will be important, and will provide a more complete and universal understanding of how division site positioning is integrated with the cell cycle and physiology of the bacterial cell.

## Conflict of Interest Statement

The authors declare that the research was conducted in the absence of any commercial or financial relationships that could be construed as a potential conflict of interest.

## References

[B1] Adams D. W. Errington J. (2009). Bacterial cell division: assembly, maintenance and disassembly of the Z ring. *Nat. Rev. Microbiol.* 7 642–653 10.1038/nrmicro2198 19680248

[B2] Atilano M. L. Pereira P. M. Yates J. Reed P. Veiga H. Pinho M. G. (2010). Teichoic acids are temporal and spatial regulators of peptidoglycan cross-linking in *Staphylococcus aureus*. *Proc. Natl. Acad. Sci. U.S.A.* 107 18991–18996 10.1073/pnas.1004304107 20944066 PMC2973906

[B3] Barak I. Wilkinson A. J. (2007). Division site recognition in *Escherichia coli* and *Bacillus subtilis*. *FEMS Microbiol. Rev.* 31 311–326 10.1111/j.1574-6976.2007.00067.x 17326815

[B4] Bates D. Kleckner N. (2005). Chromosome and replisome dynamics in E. *coli*: loss of sister cohesion triggers global chromosome movement and mediates chromosome segregation. *Cell* 121 899–911 10.1016/j.cell.2005.04.013 15960977 PMC2973560

[B5] Bernard R. Marquis K. A. Rudner D. Z. (2010). Nucleoid occlusion prevents cell division during replication fork arrest in *Bacillus subtilis*. *Mol. Microbiol.* 78 866–882 10.1111/j.1365-2958.2010.07369.x 20807205 PMC2978284

[B6] Bernhardt T. G de Boer P. A. (2005). SlmA, a nucleoid-associated, FtsZ binding protein required for blocking septal ring assembly over chromosomes in *E. coli*. *Mol. Cell* 18 555–564 10.1016/j.molcel.2005.04.012 15916962 PMC4428309

[B7] Bramkamp M. Emmins R. Weston L. Donovan C. Daniel R. A. Errington J. (2008). A novel component of the division-site selection system of *Bacillus subtilis* and a new mode of action for the division inhibitor MinCD. *Mol. Microbiol.* 70 1556–1569 10.1111/j.1365-2958.2008.06501.x 19019154

[B8] Bramkamp M van Baarle S. (2009). Division site selection in rod-shaped bacteria. *Curr. Opin. Microbiol.* 12 683–688 10.1016/j.mib.2009.10.002 19884039

[B9] Cambridge J. Blinkova A. Magnan D. Bates D. Walker J. R. (2013). A replication-inhibited un-segregated nucleoid at mid-cell blocks Z-ring formation and cell division independently of SOS and the SlmA nucleoid occlusion protein in *Escherichia coli*. *J. Bacteriol.* 196 36–49 10.1128/JB.01230-12 24142249 PMC3911132

[B10] Cho H. Mcmanus H. R. Dove S. L. Bernhardt T. G. (2011). Nucleoid occlusion factor SlmA is a DNA-activated FtsZ polymerization antagonist. *Proc. Natl. Acad. Sci. U.S.A.* 108 3773–3778 10.1073/pnas.1018674108 21321206 PMC3048121

[B11] Dajkovic A. Lan G. Sun S. X. Wirtz D. Lutkenhaus J. (2008). MinC spatially controls bacterial cytokinesis by antagonizing the scaffolding function of FtsZ. *Curr. Biol.* 18 235–244 10.1016/j.cub.2008.01.042 18291654

[B12] De Boer P. A. (2010). Advances in understanding *E. coli* cell fission. *Curr. Opin. Microbiol.* 13 730–737 10.1016/j.mib.2010.09.015 20943430 PMC2994968

[B13] Gilbo C. M. Beaton C. D. Coles N. W. (1967). Electron microscopy of the lysis of *Staphylococcus aureus* cell walls by aeromonas lytic factor. *J. Bacteriol.* 93 1972–1975 6025309 10.1128/jb.93.6.1972-1975.1967 PMC276717

[B14] Harry E. Monahan L. Thompson L. (2006). Bacterial cell division: the mechanism and its precision. *Int. Rev. Cytol.* 253 27–94 10.1016/S0074-7696(06)53002-5 17098054

[B15] Harry E. J. Rodwell J. Wake R. G. (1999). Co-ordinating DNA replication with cell division in bacteria: a link between the early stages of a round of replication and mid-cell Z ring assembly. *Mol. Microbiol.* 33 33–40 10.1046/j.1365-2958.1999.01439.x 10411721

[B16] Higgins M. L. Shockman G. D. (1970). Model for cell wall growth of *Streptococcus faecalis*. *J. Bacteriol.* 101 643–648 4984078 10.1128/jb.101.2.643-648.1970 PMC284952

[B17] Hu Z. L. Mukherjee A. Pichoff S. Lutkenhaus J. (1999). The MinC component of the division site selection system in *Escherichia coli* interacts with FtsZ to prevent polymerization. *Proc. Natl. Acad. Sci. U.S.A.* 96 14819–14824 10.1073/pnas.96.26.14819 10611296 PMC24731

[B18] Kiekebusch D. Michie K. A. Essen L. O. Lowe J. Thanbichler M. (2012). Localized dimerization and nucleoid binding drive gradient formation by the bacterial cell division inhibitor MipZ. *Mol. Cell* 46 245–259 10.1016/j.molcel.2012.03.004 22483621 PMC3355305

[B19] Land A. D. Tsui H.-C. T. Kocaoglu O. Vella S. A. Shaw S. L. Keen S. K. (2013). Requirement of essential Pbp2x and GpsB for septal ring closure in *Streptococcus pneumoniae* D39. *Mol. Microbiol.* 90 939–955 10.1111/mmi.12408 24118410 PMC4120849

[B20] Leisch N. Verheul J. Heindl N. R. Gruber-Vodicka H. R. Pende N. Den Blaauwen T. (2012). Growth in width and FtsZ ring longitudinal positioning in a gammaproteobacterial symbiont. *Curr. Biol.* 22 R831–R832 10.1016/j.cub.2012.08.033 23058799

[B21] Lenz P. Sogaard-Andersen L. (2011). Temporal and spatial oscillations in bacteria. *Nat. Rev. Microbiol.* 9 565–577 10.1038/nrmicro2612 21760621

[B22] Lutkenhaus J. (2007). Assembly dynamics of the bacterial MinCDE system and spatial regulation of the Z ring. *Annu. Rev. Biochem.* 76 14.11–14.24 10.1146/annurev.biochem.75.103004.142652 17328675

[B23] Lutkenhaus J. (2012). The ParA/MinD family puts things in their place. *Trends Microbiol.* 20 411–418 10.1016/j.tim.2012.05.002 22672910 PMC3436946

[B24] Lutkenhaus J. Pichoff S. Du S. (2012). Bacterial cytokinesis: from Z ring to divisome. *Cytoskeleton (Hoboken)* 69 778–790 10.1002/cm.21054 22888013 PMC3931253

[B25] Margolin W. (2000). Themes and variations in prokaryotic cell division. *FEMS Microbiol. Rev.* 24 531–548 10.1111/j.1574-6976.2000.tb00554.x 10978550

[B26] Margolin W. (2005). FtsZ and the division of prokaryotic cells and organelles. *Nat. Rev. Mol. Cell Biol.* 6 862–871 10.1038/nrm1745 16227976 PMC4757588

[B27] Massidda O. Nováková L. Vollmer W. (2013). From models to pathogens: how much have we learned about *Streptococcus pneumoniae* cell division? *Environ. Microbiol.* 15 3133–3157 10.1111/1462-2920.12189 23848140

[B28] Matias V. R. Beveridge T. J. (2007). Cryo-electron microscopy of cell division in *Staphylococcus aureus* reveals a mid-zone between nascent cross walls. *Mol. Microbiol.* 64 195–206 10.1111/j.1365-2958.2007.05634.x 17376082

[B29] Migocki M. D. Freeman M. K. Wake R. G. Harry E. J. (2002). The Min system is not required for precise placement of the midcell Z ring in *Bacillus subtilis*. *EMBO Rep.* 3 1163–1167 10.1093/embo-reports/kvf233 12446561 PMC1308329

[B30] Monahan L. G. Harry E. J. (2013). Identifying how bacterial cells find their middle: a new perspective. *Mol. Microbiol.* 87 231–234 10.1111/mmi.12114 23190137

[B31] Moriya S. Rashid R. A. Rodrigues C. D. Harry E. J. (2010). Influence of the nucleoid and the early stages of DNA replication on positioning the division site in *Bacillus subtilis*. *Mol. Microbiol.* 76 634–647 10.1111/j.1365-2958.2010.07102.x 20199598

[B32] Nevo-Dinur K. Govindarajan S. Amster-Choder O. (2012). Subcellular localization of RNA and proteins in prokaryotes. *Trends Genet.* 28 314–322 10.1016/j.tig.2012.03.008 22521614

[B33] Patrick J. E. Kearns D. B. (2008). MinJ (YvjD) is a topological determinant of cell division in *Bacillus subtilis*. *Mol. Microbiol.* 70 1166–1179 10.1111/j.1365-2958.2008.06469.x 18976281

[B34] Pinho M. G. Kjos M. Veening J.-W. (2013). How to get (a)round: mechanisms controlling growth and division of coccoid bacteria. *Nat. Rev. Microbiol.* 11 601–614 10.1038/nrmicro3088 23949602

[B35] Ramirez-Arcos S. Szeto J. Beveridge T. Victor C. Francis F. Dillon J. (2001). Deletion of the cell-division inhibitor MinC results in lysis of *Neisseria gonorrhoeae*. *Microbiology* 147 225–237 11160816 10.1099/00221287-147-1-225

[B36] Ramirez-Arcos S. Szeto J. Dillon J. A. Margolin W. (2002). Conservation of dynamic localization among MinD and MinE orthologues: oscillation of *Neisseria gonorrhoeae* proteins in *Escherichia coli*. *Mol. Microbiol.* 46 493–504 10.1046/j.1365-2958.2002.03168.x 12406224

[B37] Regamey A. Harry E. J. Wake R. G. (2000). Mid-cell Z ring assembly in the absence of entry into the elongation phase of the round of replication in bacteria: co-ordinating chromosome replication with cell division. *Mol. Microbiol.* 38 423–434 10.1046/j.1365-2958.2000.02130.x 11069667

[B38] Rodrigues C. D. Harry E. J. (2012). The Min system and nucleoid occlusion are not required for identifying the division site in *Bacillus subtilis* but ensure its efficient utilization. *PLoS Genet.* 8:e1002561 10.1371/journal.pgen.1002561 PMC3310732 22457634

[B39] Rothfield L. Taghbalout A. Shih Y. L. (2005). Spatial control of bacterial division-site placement. *Nat. Rev. Microbiol.* 3 959–968 10.1038/nrmicro1290 16322744

[B40] Rudner D. Z. Losick R. (2010). Protein subcellular localization in bacteria. *Cold Spring Harb. Perspect. Biol.* 2 a000307 10.1101/cshperspect.a000307 PMC2845201 20452938

[B41] Schlag M. Biswas R. Krismer B. Kohler T. Zoll S. Yu W. (2010). Role of staphylococcal wall teichoic acid in targeting the major autolysin Atl. *Mol. Microbiol.* 75 864–873 10.1111/j.1365-2958.2009.07007.x 20105277

[B42] Shapiro L. Mcadams H. H. Losick R. (2009). Why and how bacteria localize proteins. *Science* 326 1225–1228 10.1126/science.1175685 19965466 PMC7531253

[B43] Singh B. Nitharwal R. G. Ramesh M. Pettersson B. M. Kirsebom L. A. Dasgupta S. (2013). Asymmetric growth and division in *Mycobacterium* spp.: compensatory mechanisms for non-medial septa. *Mol. Microbiol.* 88 64–76 10.1111/mmi.12169 23387305

[B44] Smits W. K. Goranov A. I. Grossman A. D. (2010). Ordered association of helicase loader proteins with the *Bacillus subtilis* origin of replication in vivo. *Mol. Microbiol.* 75 452–461 10.1111/j.1365-2958.2009.06999.x 19968790 PMC2992960

[B45] Szeto J. Ramirez-Arcos S. Raymond C. Hicks L. D. Kay C. M Dillon J.-A. R. (2001). Gonococcal MinD affects cell division in *Neisseria gonorrhoeae* and *Escherichia coli* and exhibits a novel self-interaction. *J. Bacteriol.* 183 6253–6264 10.1128/JB.183.21.6253-6264.2001 11591668 PMC100108

[B46] Thanbichler M. (2009). Spatial regulation in *Caulobacter crescentus*. *Curr. Opin. Microbiol.* 12 715–721 10.1016/j.mib.2009.09.013 19854671

[B47] Thanbichler M. Shapiro L. (2006). MipZ, a spatial regulator coordinating chromosome segregation with cell division in Caulobacter. *Cell* 126 147–162 10.1016/j.cell.2006.05.038 16839883

[B48] Tonthat N. K. Arold S. T. Pickering B. F. Van Dyke M. W. Liang S. Lu Y. (2011). Molecular mechanism by which the nucleoid occlusion factor, SlmA, keeps cytokinesis in check. *EMBO J.* 30 154–164 10.1038/emboj.2010.288 21113127 PMC3020112

[B49] Tonthat N. K. Milam S. L. Chinnam N. Whitfill T. Margolin W. Schumacher M. A. (2013). SlmA forms a higher-order structure on DNA that inhibits cytokinetic Z-ring formation over the nucleoid. *Proc. Natl. Acad. Sci. U.S.A.* 110 10586–10591 10.1073/pnas.1221036110 23754405 PMC3696773

[B50] Touhami A. Jericho M. H. Beveridge T. J. (2004). Atomic force microscopy of cell growth and division in Staphylococcus aureus. *J. Bacteriol.* 186 3286–3295 10.1128/JB.186.11.3286-3295.2004 15150213 PMC415778

[B51] Traag B. A van Wezel G. P. (2008). The SsgA-like proteins in actinomycetes: small proteins up to a big task. *Antonie Van Leeuwenhoek* 94 85–97 10.1007/s10482-008-9225-3 18273689 PMC2440963

[B52] Treuner-Lange A. Aguiluz K. Van Der Does C. Gomez-Santos N. Harms A. Schumacher D. (2013). PomZ, a ParA-like protein, regulates Z-ring formation and cell division in *Myxococcus xanthus*. *Mol. Microbiol.* 87 235–253 10.1111/mmi.12094 23145985

[B53] Turner R. D. Ratcliffe E. C. Wheeler R. Golestanian R. Hobbs J. K. Foster S. J. (2010). Peptidoglycan architecture can specify division planes in *Staphylococcus aureus*. *Nat. Commun.* 1 26 10.1038/ncomms1025 20975691

[B54] Tzagoloff H. Novick R. (1977). Geometry of cell division in *Staphylococcus aureus*. *J. Bacteriol.* 129 343–350 830642 10.1128/jb.129.1.343-350.1977 PMC234932

[B55] Veiga H. Jorge A. M. Pinho M. G. (2011). Absence of nucleoid occlusion effector Noc impairs formation of orthogonal FtsZ rings during *Staphylococcus aureus* cell division. *Mol. Microbiol.* 80 1366–1380 10.1111/j.1365-2958.2011.07651.x 21477126

[B56] Westling-Häggström B. Elmros T. Normark S. Winblad B. (1977). Growth pattern and cell division in *Neisseria gonorrhoeae*. *J. Bacteriol.* 129 333–342 401495 10.1128/jb.129.1.333-342.1977 PMC234931

[B57] Wheeler R. Mesnage S. Boneca I. G. Hobbs J. K. Foster S. J. (2011). Super-resolution microscopy reveals cell wall dynamics and peptidoglycan architecture in ovococcal bacteria. *Mol. Microbiol.* 82 1096–1109 10.1111/j.1365-2958.2011.07871.x 22059678

[B58] Willemse J. Borst J. W. De Waal E. Bisseling T Van Wezel G. P. (2011). Positive control of cell division: FtsZ is recruited by SsgB during sporulation of Streptomyces. *Genes Dev.* 25 89–99 10.1101/gad.600211 21205868 PMC3012939

[B59] Wu L. J. Errington J. (2004). Coordination of cell division and chromosome segregation by a nucleoid occlusion protein in *Bacillus subtilis*. *Cell* 117 915–925 10.1016/j.cell.2004.06.002 15210112

[B60] Wu L. J. Errington J. (2012). Nucleoid occlusion and bacterial cell division. *Nat. Rev. Microbiol.* 10 8–12 10.1038/nrmicro2671 22020262

[B61] Wu L. J. Franks A. H. Wake R. G. (1995). Replication through the terminus region of the *Bacillus subtilis* chromosome is not essential for the formation of a division septum that partitions the DNA. *J. Bacteriol.* 177 5711–5715 7559364 10.1128/jb.177.19.5711-5715.1995 PMC177386

[B62] Wu L. J. Ishikawa S. Kawai Y. Oshima T. Ogasawara N. Errington J. (2009). Noc protein binds to specific DNA sequences to coordinate cell division with chromosome segregation. *EMBO J.* 28 1940–1952 10.1038/emboj.2009.144 19494834 PMC2711181

[B63] Yu W. Herbert S. Graumann P. L Götz F. (2010). Contribution of SMC (structural maintenance of chromosomes) and SpoIIIE to chromosome segregation in staphylococci. *J. Bacteriol.* 192 4067–4073 10.1128/JB.00010-10 20525833 PMC2916368

[B64] Yu X. C. Margolin W. (1999). FtsZ ring clusters in min and partition mutants: role of both the Min system and the nucleoid in regulating FtsZ ring localization. *Mol. Microbiol.* 32 315–326 10.1046/j.1365-2958.1999.01351.x 10231488

[B65] Zapun A. Vernet T. Pinho M. G. (2008). The different shapes of cocci. *FEMS Microbiol. Rev.* 32 345–360 10.1111/j.1574-6976.2007.00098.x 18266741

